# Lesion Eccentricity Plays a Key Role in Determining the Pressure Gradient of Serial Stenotic Lesions: Results from a Computational Hemodynamics Study

**DOI:** 10.1007/s00270-024-03708-x

**Published:** 2024-04-02

**Authors:** L. van de Velde, E. Groot Jebbink, K. Jain, M. Versluis, M. M. P. J. Reijnen

**Affiliations:** 1 Multi-Modality Medical Imaging M3i Group, TechMed Centre, University of Twente, Drienerlolaan 5, 7522 NB Enschede, The Netherlands; 2 Department of Surgery, Rijnstate, Arnhem, The Netherlands; 3 Physics of Fluids Group, TechMed Centre, University of Twente, Enschede, The Netherlands; 4 Department of Thermal and Fluid Engineering, University of Twente, Enschede, The Netherlands

**Keywords:** Serial stenosis, Eccentricity, Pressure gradient, Fractional flow reserve, Hemodynamics

## Abstract

**Purpose:**

In arterial disease, the presence of two or more serial stenotic lesions is common. For mild lesions, it is difficult to predict whether their combined effect is hemodynamically significant. This study assessed the hemodynamic significance of idealized serial stenotic lesions by simulating their hemodynamic interaction in a computational flow model.

**Materials and Methods:**

Flow was simulated with SimVascular software in 34 serial lesions, using moderate (15 mL/s) and high (30 mL/s) flow rates. Combinations of one concentric and two eccentric lesions, all 50% area reduction, were designed with variations in interstenotic distance and in relative direction of eccentricity. Fluid and fluid–structure simulations were performed to quantify the combined pressure gradient.

**Results:**

At a moderate flow rate, the combined pressure gradient of two lesions ranged from 3.8 to 7.7 mmHg, which increased to a range of 12.5–24.3 mmHg for a high flow rate. Eccentricity caused an up to two-fold increase in pressure gradient relative to concentric lesions. At a high flow rate, the combined pressure gradient for serial eccentric lesions often exceeded the sum of the individual lesions. The relative direction of eccentricity altered the pressure gradient by 15–25%. The impact of flow pulsatility and wall deformability was minor.

**Conclusion:**

This flow simulation study revealed that lesion eccentricity is an adverse factor in the hemodynamic significance of isolated stenotic lesions and in serial stenotic lesions. Two 50% lesions that are individually non-significant can combine more often than thought to hemodynamic significance in hyperemic conditions.

**Graphical Abstract:**

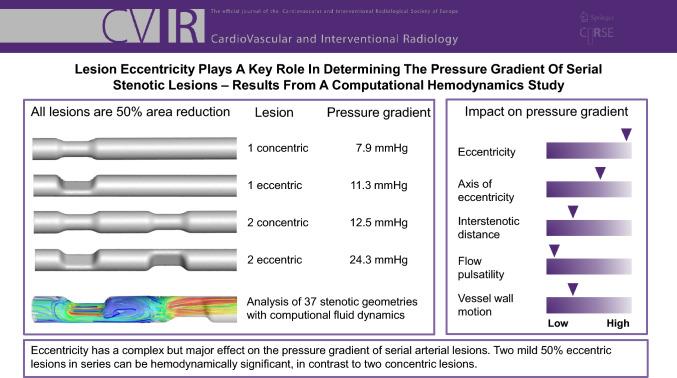

**Supplementary Information:**

The online version contains supplementary material available at 10.1007/s00270-024-03708-x.

## Introduction

In clinical practice, the severity of arterial stenotic lesions is quantified non-invasively by anatomic grading through imaging or by measuring blood velocities using duplex ultrasonography [1]. These approaches have a reasonable correspondence with invasively measured pressure gradients over a stenosis [2], but their application for defining significance of multilevel or tandem stenotic lesions is limited [3]. For calculating systolic velocity ratios, a reference upstream peak systolic velocity for the downstream stenosis is unreliable, as this velocity may be elevated by the upstream stenosis [4]. Moreover, the stenoses may hemodynamically interact if they are in close proximity [5], making it unclear if and when two stenoses of borderline significance add up to a combined hemodynamic significance.

Serial stenotic lesions are common and have been reported in 41% of femoropopliteal arteries [4] subject to endovascular treatment and in 29% of coronary arteries subject to angiography [6]. For coronary stenoses, a measurement of the translesional pressure gradient during hyperemia is commonly taken. From this measurement, the fractional flow reserve (FFR) can be derived, which in combination with the measurement of wedge pressure can be used to estimate the individual severity of both lesions [7]. FFR measurements are invasive, however, and for peripheral regions, the materials and equipment required for these measurements are often not available. It would therefore be beneficial to have an understanding for when two lesions combined lead to hemodynamic significance.

Previous studies have addressed the theoretical [8] and practical aspects of pressure gradients of single concentric and eccentric stenosis [9, 10], as well as the combined pressure gradient of serial concentric lesions [5, 11, 12]. The pressure gradient of a single lesion is determined primarily by the stenosis shape, the flow velocity, and the Reynolds number, defined as the ratio of the vessel diameter and flow velocity relative to the fluid viscosity [8]. Whether the individual gradients of two concentric lesions are mutually additive depends on whether the distal stenosis is close enough to the proximal stenosis to interfere with normalization of the post-stenotic jet [8, 11]. This normalization distance will depend on the Reynolds number and stenotic shape and severity of the proximal stenosis. For interstenotic distances smaller than this normalization length, e.g., less than 10 diameters for non-turbulent flow [5], the total pressure gradient becomes less than the sum of the two individual stenoses, due to the convective energy losses of the first stenosis being limited by the second. For severe stenotic lesions (> 90%) with turbulent flow, the effect of two isolated regions has been reported to linearly add up when the interstenotic distance is more than four diameters [8]. Drawback of these studies is the fact that only serial concentric lesions were assessed and steady flow and rigid walls were assumed.

The convective energy loss of eccentric stenoses is more complex, which may produce unforeseen interactions between multiple eccentric stenoses. The purpose of this study is to evaluate hemodynamic significance of serial stenotic lesions, by simulating a range of combinations of both eccentric and concentric stenoses. Variety in eccentric shape and distance between the proximal and distal lesions, in addition to differences in flow rate and the effect of flow pulsatility and wall deformability, will be investigated.

## Methods

### Stenotic Flow Models

**Fig. 1:**
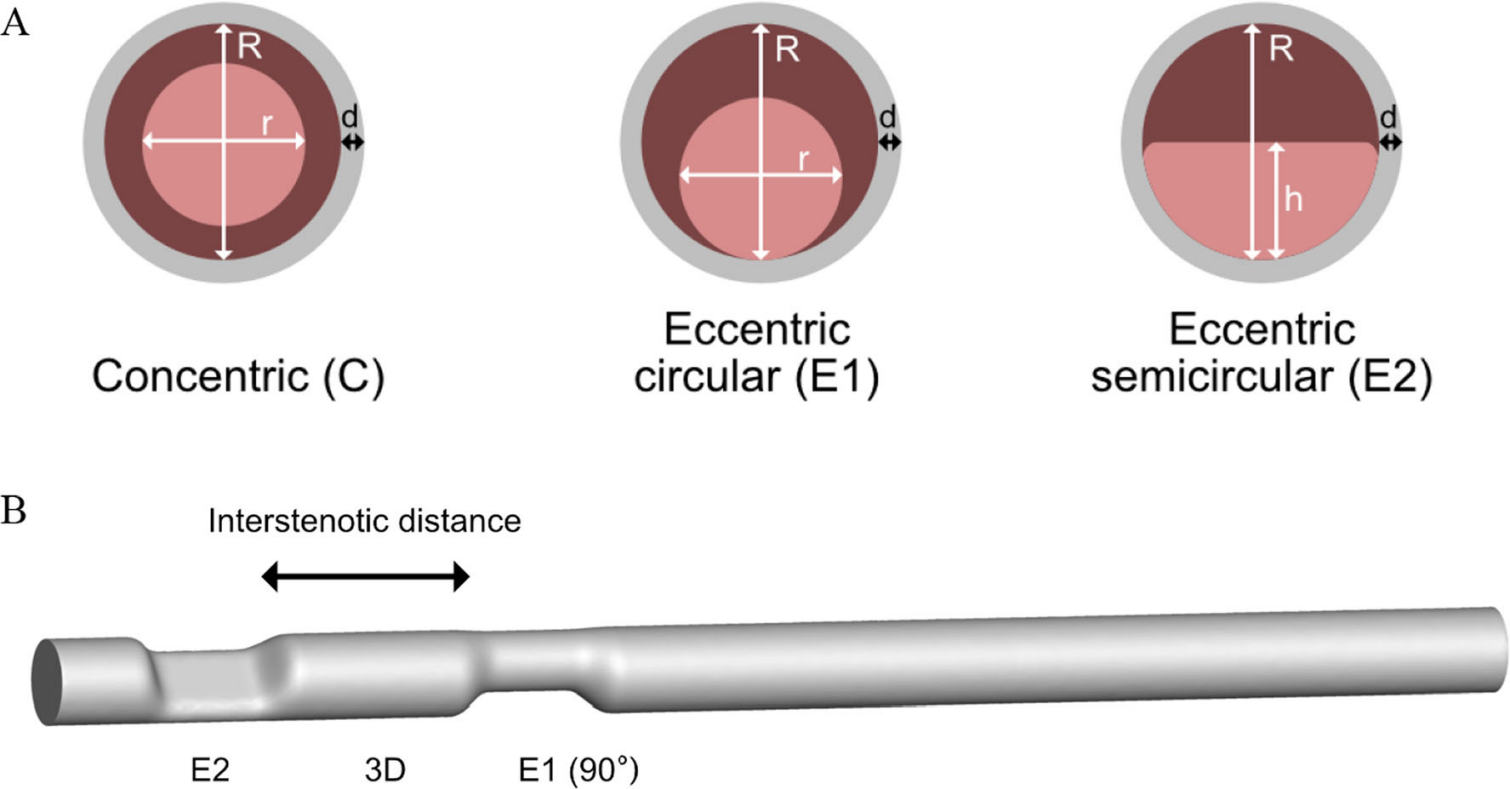
Stenotic geometries; **A** a cross-section of the three modeled stenotic geometries. *R* = 6 mm, *r* = $\frac{6}{\sqrt{2}}$ mm, *h* = 3 mm, *d* = 0.3 mm. **B** The E2–E1-3D-90° model, with an E2 proximal stenosis, E1 distal stenosis, an interstenotic distance of three diameters, and a relative eccentricity rotation of 90°

Blood flow was simulated with the open-source SimVascular software [13], capable of simulating pulsatile flow and vessel wall motion. Flow was simulated through three shapes of stenotic lesions (Fig. 1A): a concentric lesion (C), an eccentric circular lesion (E1), and an eccentric semicircular lesion (E2). These geometries were investigated as isolated lesions in a previous study [9]. A nominal 6-mm diameter was chosen for the models, which reflects the average human diameter of the superficial femoral artery. All three lesion cross-sections corresponded to a 50% area reduction, and the C lesion was slightly (0.01 mm) offset from the centerline to exclude an unrealistic purely axisymmetric solution. The lesions were investigated as a single lesion and through combinations of two stenoses with varying interstenotic length: three non-stenotic reference diameters (3D = 18 mm) and six diameters (6D = 36 mm) (Fig. 1B). For the 3D and 6D scenarios, nine combinations of the first and the second stenoses shape were possible. In addition, in case of two eccentric stenoses, the rotation angle can be varied from 0 to 180°. For these cases, rotation angles of 0, 90, and 180 degrees were chosen, yielding 17 unique combinations for the 3D and 6D distances each. In combination with the single stenoses, this resulted in a total of 37 geometries. The geometries were designed in SolidWorks 2022 (Dassault Systèmes, Vélizy-Villacoublay, France). The lofting operation was applied to smoothly bridge healthy and stenotic parts, with start and end constraints set as ‘normal to profile’ with a direction vector length of 1 mm.

### Rigid Wall Simulation

The open-source finite element-based SimVascular software [13] was used to mesh the inner geometry with tetrahedral elements, combined with a prismatic boundary layer. Blood was considered as a Newtonian fluid with a dynamic viscosity of 3.5 mPa.s and a density of 1059 kg.m^3^, and the vessel walls were modeled as mechanically rigid. A parabolic profile at a steady flow rates of 15 mL/s and 30 mL/s was set. The 15 mL/s corresponded to the mean flow rate in the superficial femoral artery that we measured in seven healthy volunteers (four males, age 20–30 years). This flow rate in a 6-mm artery is characterized by a Reynolds number of 933. The 50%-area stenoses doubled the mean velocity, yielding a Reynolds number downstream of the lesions of roughly 1800. For this flow rate, the stenoses under investigation are individually of subclinical significance (pressure gradient < 5 mmHg), and turbulent effects play a minor role, if any. The higher 30 mL/s flow rate was simulated to investigate whether the stenotic interactions would appreciably change during increased blood flow. At the outlet, a resistance boundary condition was prescribed that yielded a distal pressure of 97 mmHg.

To assess the effect of flow pulsatility, unsteady simulations were performed for which a sine wave with an amplitude of 5 mL/s and a frequency of 1 Hz was added to the steady 15-mL/s flow rate, yielding a pulsatile flow between 10 and 20 mL/s. For this flow rate, a Windkessel RCR outflow condition [13] was set with the capacitance value tuned to reproduce a pulse pressure of 40 mmHg.

### Deformable wall simulation

To investigate whether wall motion could meaningfully impact the flow dynamics in serial stenoses, simulations of fluid–structure interactions for a subset of the models were performed. Two methodologies were employed: the Coupled Momentum Method (CMM) [14] and Arbitrary Lagrangian–Eulerian (ALE) method [15]. The CMM method approximates the vessel wall as a thin linearly elastic membrane, which constitutes an efficient approach for fluid–structure interaction (FSI) when significant bending is absent [14]. For the ALE method, the vessel wall was separately meshed, and its nonlinear structural mechanics was fully evaluated using a monolithic approach implemented in the separate SimVascular FSI solver [15].

Elasticity of the vessel wall was set to 40 MPa with a constant wall thickness of 0.3 mm (10% of the lumen radius). These values correspond to a physiologic wall displacement by pulse pressure of about 10% of the radius in a healthy artery [14]. For the ALE method, the vessel outer wall was pre-stressed [16] and kept in place with external tissue support with parameters that mimicked external tissue support of the superficial femoral artery by its surrounding muscle tissue [17].

The online supplemental information provides further details on the mesh convergence, the vessel wall mesh, the constitutive model used for the vessel wall, and the initialization and boundary conditions for the ALE simulation.

## Results

**Fig. 2:**
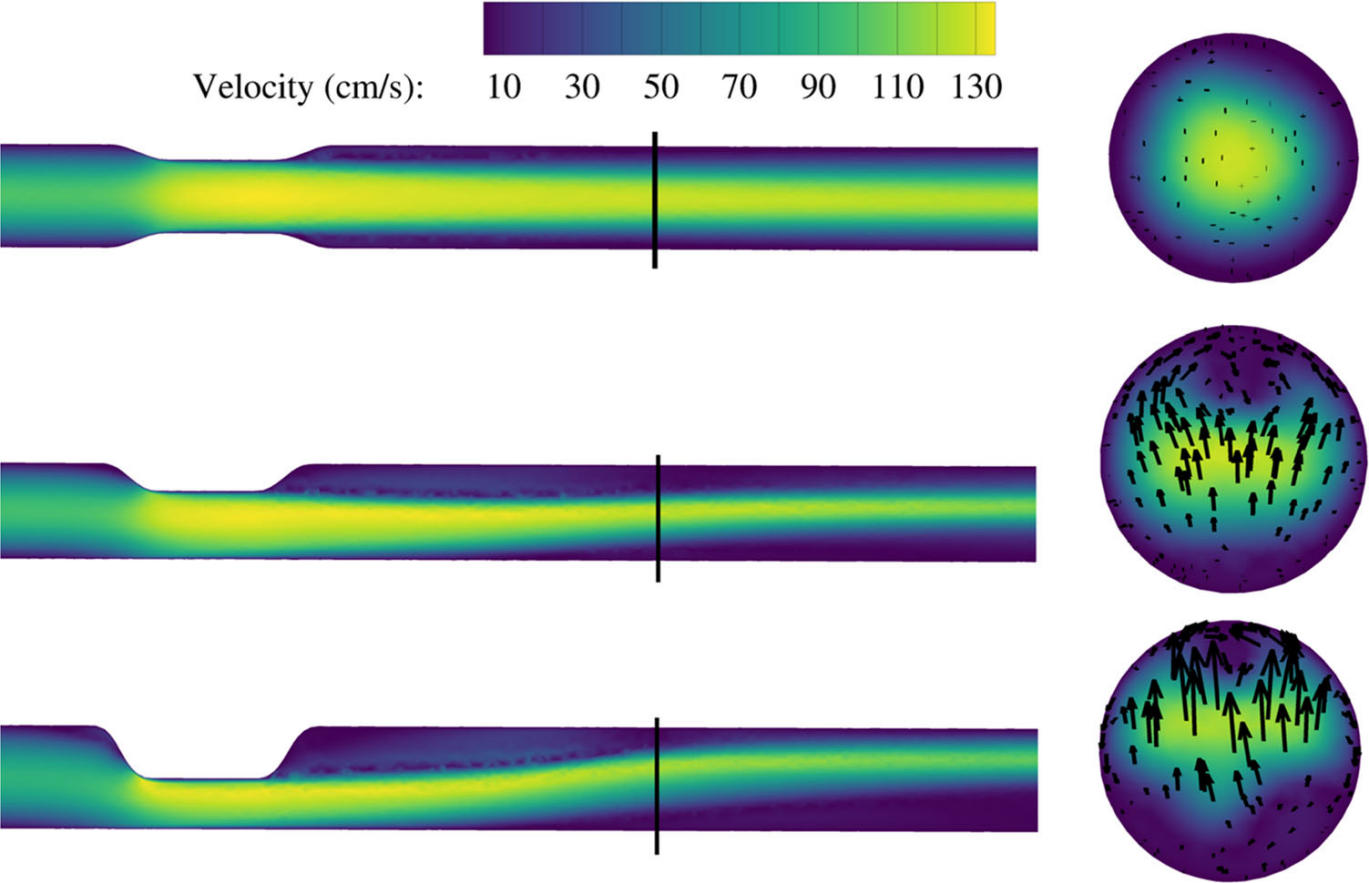
Velocity contours for single stenotic lesions for a flow rate of 15 mL/s. Left: longitudinal view, right: cross-sectional view at the location of the black line. Upper: concentric (C); middle: eccentric circular (E1); lower: eccentric semicircular (E2). The secondary flow vectors are displayed in the right cross-sections, with a maximum of 9.2 cm/s for the E2 model

For the single stenosis, the pressure gradient at 15 mL/s equaled 2.3 mmHg for the concentric lesion (C), 2.9 mmHg for the circular eccentric lesion (E1), and 4.1 mmHg for the semicircular eccentric lesion (E2). The velocity contours (Fig. 2) show that peak velocity for all three shapes were of similar order (140 cm/s). The E1 stenosis generated one recirculation area in the post-stenotic region, downstream of which parabolic flow reestablished. The E2 stenosis, in contrast, induced a second area of stagnant flow further downstream at the opposite wall of the first recirculation zone.

For the serial stenotic shapes, the observed pressure gradients for the baseline and high flow rate are listed in Table 1. Several trends can be observed. For a moderate flow rate (15 mL/s), the pressure gradient of two stenotic lesions never exceeded the sum of their parts in any of the cases. For the high flow rate (30 mL/s), there were many combinations where the combined pressure gradient exceeded the sum of the individual lesions by up to 10%. This was only the case for combinations involving an E2 stenosis. Second, an increase in the distance between the two stenoses was in most cases associated with an increased pressure gradient, except for some geometries with a distal eccentric stenosis, especially those with a 90° rotation angle. For the E1–E2 cases with a 90° rotation angle, the pressure gradient was higher for a 3D interstenotic compared to a 6D distance. Furthermore, a 90° rotation between two eccentric stenoses caused higher pressure gradients compared to 0° and 180° for the baseline flow rate, with some exceptions to this rule for the high flow rate.

**Table 1:** Pressure gradients for steady flow

Proximal stenosis	Distal stenosis	Interstenotic distance	Rotation angle	$\Delta P$- 15 mL/s, mmHg	$\Delta P$- 30 mL/s, mmHg
C	–	–	–	2.3	7.9
E1	–	–	–	2.9	8.3
E2	–	–	–	4.1	11.3
C	C	3D	–	3.8	12.5
	C	6D	–	4.1	13.4
C	E1	3D	–	4.2	14.0
	E1	6D	–	4.4	13.4
C	E2	3D	–	6.2	19.0
	E2	6D	–	5.7	18.6
E1	C	3D	–	4.4	12.7
	C	6D	–	4.6	14.2
E1	E1	3D	0°	4.2	12.6
	E1	3D	90°	4.8	14.2
	E1	3D	180°	4.7	13.8
E1	E1	6D	0°	4.7	14.7
	E1	6D	90°	5.0	14.5
	E1	6D	180°	4.7	14.0
E1	E2	3D	0°	5.6	17.3
	E2	3D	90°	6.7	20.7
	E2	3D	180°	5.5	16.9
E1	E2	6D	0°	6.0	18.9
	E2	6D	90°	6.6	20.3
	E2	6D	180°	5.9	16.4
E2	C	3D	–	5.6	15.8
	C	6D	–	6.0	18.2
E2	E1	3D	0°	5.5	17.0
	E1	3D	90°	6.1	18.7
	E1	3D	180°	5.9	18.5
E2	E1	6D	0°	6.4	20.6
	E1	6D	90°	6.4	19.9
	E1	6D	180°	6.2	18.7
E2	E2	3D	0°	6.3	19.2
	E2	3D	90°	7.6	24.3
	E2	3D	180°	6.2	20.3
E2	E2	6D	0°	7.1	24.8
	E2	6D	90°	7.7	23.8
	E2	6D	180°	7.0	22.4

*C* concentric*, E*1 eccentric circular*, E*2 eccentric semicircular*, D* diameter (6 mm)

**Fig. 3:**
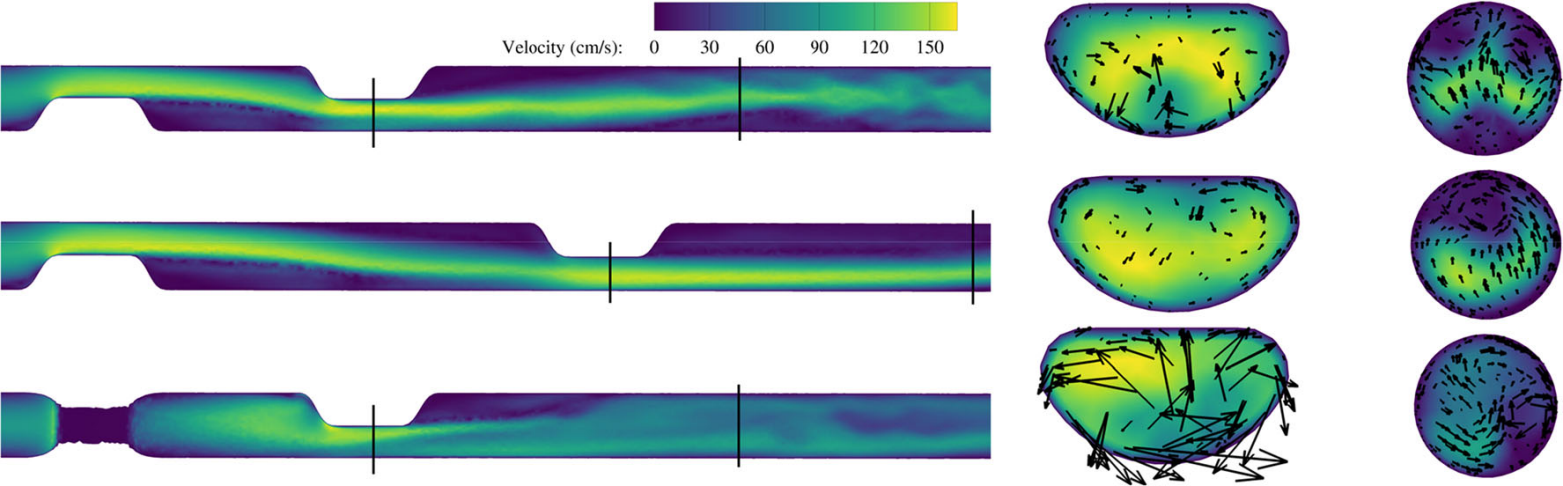
Velocity contours for three eccentric semicircular (E2) stenotic models at a flow rate of 15 mL/s. Upper: E2–E2-3D-180°; middle: E2–E2-6D-180°; lower: E2–E2-3D-90°. Secondary flow vectors for two cross-sections are displayed, with a maximum of 58 cm/s in the distal stenosis of the lower model

The velocity contours for a selection of double semicircular eccentric lesions are plotted in Fig. 3. In the upper plot with a 3D interstenotic distance, only one distinct area of flow recirculation was present in the interstenotic region. In the larger 6D interstenotic area for the middle plot, two distinct areas of flow recirculation were present. The lower plot highlights the altered flow dynamics for a 90° rotation model, where strong secondary flow structures were present in the distal stenosis that caused a more abrupt and chaotic breakdown of the post-stenotic jet with an associated increase in the pressure gradient.

The impact of pulsatile flow and wall deformability on the pressure gradient was investigated for several models and is listed in Table 2. Flow pulsatility in rigid simulations did not meaningfully alter the recirculation zones, and the mean pressure gradient for pulsatile flow was only slightly higher than the corresponding steady flow rate for most cases. The simulations with wall deformability demonstrated categorically different results depending on the applied simulation method. For most models, a substantial increase in the pressure gradient was present for the CMM method, yet only minor increases were observed for the ALE method. An exception is the E2–E2-3D-90° model, which demonstrated wall motion that translated the distal E2 stenosis 4.4 mm from its baseline positioning, straightening the trajectory of the flow and reducing the areas of recirculation and the pressure gradient. The ALE simulations with a thicker wall at the stenosis (constant outer vessel diameter, see online supplementary material) led to slightly less wall motion but had minimal impact on the pressure gradient and velocity field.

**Table 2:** Pressure gradients (mmHg) for pulsatile flow in rigid wall and deformable wall models

Model	Rigid, steady flow	Rigid, pulsatile flow	Deformable, CMM	Deformable, ALE
C–C-3D	3.9	4.0	7.4	4.6
C–C-6D	4.2	4.3	7.2	4.8
C–E1-6D	4.4	4.8	5.2	5.4
E2–E2-3D-90°	7.6	7.9	9.0	4.4

*CMM* Coupled momentum method*, ALE* Arbitrary Lagrangian–Eulerian method

The different manifestations of wall motion and the consequences for the velocity field are presented for the C–C-6D model in Video 1. The CMM method showed a high-frequent axial buckling [18] motion of the wall, coupled with a fluttering motion of the post-stenotic jet downstream of the proximal and distal stenoses. This motion was associated with a 48-Hz oscillatory mode in the pressure field and led to a strong increase in the pressure gradient. This high-frequent oscillatory behavior of the wall and the fluid motion was not present in the ALE method, which demonstrated a slightly increased pressure gradient compared to a rigid wall. The fluid motion in the ALE method was largely comparable to the rigid wall simulations, showing asymmetric development of the jet downstream of the distal stenosis associated with a subtle asymmetric wall motion. The pressure wave in the ALE simulation was delayed in phase and reduced in amplitude relative to the rigid wall simulation. This reflected the dampening of the pressure wave by wall compliance. Furthermore, the intra- and post-stenotic jet in the rigid simulation demonstrated a slightly broadened spatial profile with a lower peak velocity relative to the ALE simulation.

## Discussion

The decision whether to treat two subclinical stenoses is difficult in clinical practice, as it is unclear what the threshold for combined hemodynamic significance is. Traditional measures like the percentual diameter or area reduction or the Doppler peak systolic velocity ratio do not reflect the additive effect but only assess the severity of the most severe stenosis [3]. This simulation study demonstrated that eccentricity is a key element in the hemodynamic significance of both single and serial 50%-area lesions. Two unfavorably arranged semicircular eccentric lesions demonstrated a gradient of 24 mmHg, relative to 12 mmHg for two concentric lesions of equal area reduction. Furthermore, for two eccentric lesions, the combined pressure gradient at high flow rates was often found to exceed the sum of its two isolated lesions, highlighting the adverse impact of eccentricity in serial lesions. In symptomatic patients with one or more subcritical (e.g., < 75% area) eccentric lesions, this suggests that treatment of the lesion(s) may improve symptoms.

The role of eccentricity was complex and flow rate-dependent. This has also been demonstrated for single stenotic lesions, where eccentricity did not increase the pressure gradient for low flow rates (Reynolds number 10–1000) [9], but with two-fold increases for moderate flow rates (Reynolds number > 1000) [10]. In this study, the impact of eccentricity also increased for higher flow rates, which can explain the 22.7% discordance between resting gradient and hyperemic gradient classification of serial lesions [12]. For two lesions that involved the most eccentric E2 lesion, the combined pressure gradient exceeded the sum of its two isolated lesions for a high flow rate, which was never the case for a moderate flow rate. This observation indicates that moderate serial eccentric lesions may combine to hemodynamic significance in exercise conditions. This could have important consequences for symptomatic patients with mild serial eccentric lesions on anatomic or duplex ultrasound evaluation [4], which may currently not be referred for treatment or for a physiologic evaluation. Eccentric lesions are very common in both the femoral (64%) [19] and the coronary arteries (45.6%) [20]. For clinical evaluation of stenotic disease, it is therefore important to appreciate the increased likelihood of hemodynamic significance of two mild lesions when they have an eccentric shape. In these cases, diagnostic thresholds are not possible with duplex ultrasound and are hard to make with CTA, and depending on the localization, an exercise ankle-brachial index or invasive pressure measurement might be needed.

Two other geometric effects that were investigated were the interstenotic distance and the relative rotation of the eccentric lesions. For most cases, an increase in the interstenotic distance was associated with increase in the pressure gradient, with some exceptions, notably  for cases with a distal E2 stenosis. These exceptions conflict with previous studies where an increase in interstenotic distance was exclusively associated with an increase in the pressure gradient [5, 11]. This discrepancy is likely explained by the importance of the inflow profile into the distal stenosis. In the E2 cases, the proximal stenosis led to inflow disturbances into the distal stenosis that increased outflow disturbances and the pressure loss of the distal stenosis. This hemodynamic interaction is similar to a previous observation [5] that a proximal 75% concentric stenosis with a distal 50% concentric stenosis caused a higher pressure gradient compared to a reverse configuration. The effects of interstenotic distance were usually below 10%, however, and without a clear trend making the impact of minor relevance for clinical practice for peripheral arteries. For coronary arteries, serial stenotic lesions with an interstenotic distance below three reference vessel diameters are treated as a single lesion in the SYNTAX I and II scores [21], which may underestimate the significance of these lesions when used for individual risk prediction. Particularly when of eccentric shape, such lesions are likely better assessed with invasive or computational physiologic evaluation.

With respect to the relative rotation of eccentric lesions, a consistent trend was present for the baseline flow where a 90° rotation caused 10–20% higher pressure gradients than for a 0° or 180° rotation. For the high flow rate, this trend was still present but less consistent. In clinical practice, the adverse 90° rotational configuration of serial eccentric lesions is difficult to assess with angiography but can be assessed on pre-operative CTA for lesions with an uncertain indication for intervention.

A two-fold increase in flow rate led to a roughly three-fold increase in the pressure gradient across the serial stenotic models. The high flow rate was considered representative of the peak flow rate during systole or the mean flow rate during exercise and led to a hemodynamically significant pressure gradient of over 20 mmHg [22] for eight of the 34 models. The three-fold increase is in line with the theoretical relation of the pressure gradient to the sum of a viscous pressure—linearly related with flow rate—and an inertial pressure loss that scales with the square of the flow rate [8].

Flow pulsatility with a single harmonic oscillation did not significantly alter the pressure gradient for non-compliant walls. The addition of wall deformability led to high-frequent flutter in the CMM method with a significantly increased energy loss. In the comparably more realistic ALE, this behavior was absent, and the flow field largely resembled the rigid wall simulations, with an increase in the pressure gradient of about 10%. An exception was formed by the E2–E2 model, in which a lumen straightening was present during systole, limiting flow recirculation and decreasing the pressure gradient. This behavior is likely unrealistic in diseased peripheral arteries, and the application of higher elasticity of external tissue support would limit vessel displacement.

In the ALE method, a thick wall, the nonlinearized kinematics, and external tissue support including viscous energy loss were modeled. These factors, absent in the presently applied CMM method [14], more realistically represent a bounded vessel wall and likely stabilized the fluid–structure interaction from the growth of the spurious oscillations. In calcified lesions, the wall motion will be decreased [23], limiting the observed effects of the simulated healthy vessel wall. For lipid-filled plaques [23] and vessel diseases that structurally weaken the vessel wall, such as fibromuscular dysplasia, the effects of wall motion may be amplified, and perhaps oscillatory fluid–structure interaction modes can be present. For multifocal fibromuscular dysplasia with a typical string-of-beads appearance, such an effect may contribute to the unexpectedly high pressure gradients that have been described in a few patient cases [24, 25].

The effects described in this study were observed for computational models of 6-mm arteries. They can reasonably be generalized to similarly shaped 50%-area stenoses in arteries of other diameters if similar velocities are present (spatiotemporal mean velocity of 53 cm/s for resting flow). This is because in the present Reynolds numbers (Re = 1000), inertial effects dominate (inertial effect accounts for > 70% of pressure gradient for a 50% stenosis at a Reynolds number of 1000), in which case the pressure gradient is mostly influenced by the stenotic area reduction and flow velocity, and less so by the Reynolds number [8]. Other factors such as systemic blood pressure have no direct effect on the pressure gradient but can have an impact through changes in the flow velocity.

### Study Limitations

An important limitation for translating the results to clinical practice of this study is that only three smooth stenotic shapes were assessed. Stenotic morphology in patients is highly variable, and especially calcified plaques are characterized by surface irregularity. For assessing flow mechanics in the variety of stenotic shapes in patients, image-based computational fluid dynamic simulations are an attractive and validated method for coronary lesions [26]. For peripheral arteries, computational fluid dynamic simulations of a patient’s geometry can furthermore be informed with a patient’s temporal flow profile obtained from duplex ultrasound [27]. Calcified plaques are difficult to quantify accurately using non-invasive imaging and may require intra-vascular ultrasonic or optical imaging [28] for accurate simulations. It would be of interest to investigate whether the observed adverse effect of eccentricity also holds for other stenotic degrees and whether a correlation between eccentricity index [20] and pressure gradient is present in patients.

Further simplifications of this study were the single harmonic flow waveform and the assumption of a Newtonian fluid model. For the infrarenal aorta and its peripheral arteries, the biphasic or triphasic flow will likely lead to more complex flow phenomena, although the mean pressure gradient may not be strongly affected. The inclusion of power-law viscosity model was previously shown to minimally alter the pressure gradient in serial stenotic lesions [11].

## Conclusions

The hemodynamic interaction between two stenotic lesions in proximity was complex, especially in case of eccentric lesions. Specific configurations of two 50% eccentric stenotic lesions led to surprisingly high pressure gradients. The pressure gradient of two eccentric lesions was up to twice as high as two similar concentric stenotic lesions and commonly exceeded the sum of the individual eccentric at a high flow rate. For most cases, the effects of pulsatile flow and wall motion were minor in comparison with lesion eccentricity. These findings suggest that symptomatic patients with two or more subcritical eccentric lesions may benefit from treatment and should ideally be evaluated with a hyperemic pressure measurement.

### Supplementary Information

Below is the link to the electronic supplementary material.

Supplementary file1 (DOCX 1303 KB)

Supplementary file 2. Video 1: Velocity contours for CC6D model with rigid wall and deformable wall simulations. Playback speed is 5x slower than real-time.

## Abbreviations

ALE: Arbitrary Lagrangian–Eulerian
CMM: Coupled momentum method
C: Concentric 50% stenosis
E1: Eccentric offset circular stenosis
E2: Semicircular eccentric stenosis
FFR: Fractional flow reserve
