# Supplementary material for: Lesion Eccentricity Plays a Key Role in Determining the Pressure Gradient of Serial Stenotic Lesions: Results from a Computational Hemodynamics Study
Source: Cardiovasc Intervent Radiol. 2024 Apr 2;47(5):533–42. doi: 10.1007/s00270-024-03708-x (PMC11074038; doi:10.1007/s00270-024-03708-x)
Supplement: Supplementary file 1 — Supplementary file1 (DOCX 1303 KB) [file 270_2024_3708_MOESM1_ESM.docx]

**SUPPLEMENTARY MATERIAL**

To “Lesion eccentricity plays a key role in determining the pressure gradient of serial stenosis lesions – a computational hemodynamics study”.

*L. van de Velde, E. Groot Jebbink, K. Jain, M. Versluis, M.M.P.J. Reijnen.*

**Expanded Methods**

This supplement discusses details regarding the mesh convergence of the simulations, as well as the modeling approach and boundary conditions for the deformable wall simulations.

For the simulations, solutions were considered converged if the combined mass and momentum residuals were lower than 10^-3^. The end point was the pressure difference between the inlet and outlet boundary. Mesh convergence was therefore assessed by doubling the mesh size until the pressure difference changed no more than 1%.

For the simulations that included vessel wall motion, a Poisson ratio of 0.49 was set and wall density was considered equal to blood density. The material constants were set as shell parameters for the CMM and as Neo-Hookean constitutive model of the vessel wall for the ALE method. For the ALE simulations two wall meshes were created. One used a similar wall mesh to the CMM method that had a constant 0.3 mm wall thickness, i.e. the vessel wall decreased in diameter at the stenosis part. For the second a wall mesh was created with an increased, variable wall thickness at the stenotic lesions, such that the outer wall diameter was constant at 6.6 mm.

To simulate in a physiologic operating range of the constitutive stress-strain relationship for the ALE simulation, pre-stress in the vessel wall corresponding to the interfacial pressure and shear stress obtained from a steady rigid wall simulation was computed and included in the wall stress tensor.[16] The inlet and outlet wall nodes were fixed in space. Second, to mimic external tissue support and obtain a stable FSI simulation, Robin boundary conditions for the vessel outer wall were set with spring and damper values equal to 12.6 kPa and 244 Pa.s, respectively. These values are representative for the external tissue support of the superficial femoral artery by its surrounding muscle tissue.[17]

# **References**

16. Hsu MC, Bazilevs Y. Blood vessel tissue prestress modeling for vascular fluidstructure interaction simulation. Finite Elem Anal Des. 2011;47(6):593–9.

17. Kang MJ, Yoo HH. In vivo viscoelastic properties of human thigh under compression estimated by experimental results obtained with pendulum test. Int J Precis Eng Manuf. 2017;18(9):1253–62.
